# Long non-coding RNA CTBP1-AS2 upregulates USP22 to promote pancreatic carcinoma progression by sponging miR-141-3p

**DOI:** 10.3892/mmr.2022.12602

**Published:** 2022-01-14

**Authors:** Mingliang Zhang, Songbo Ma, Xuzhao Li, Henghai Yu, Yizheng Tan, Jun He, Xiaoping Wei, Junming Ma

**Affiliations:** 1 Department of General Surgery, People's Hospital of Ningxia Hui Autonomous Region, Yinchuan, Ningxia Hui Autonomous Region 750021, P.R. China; 2 Department of Hepatopancreatobiliary Surgery, The 2nd Hospital of Kunming Medical University, Kunming, Yunnan 650101, P.R. China

**Keywords:** pancreatic carcinoma, long non-coding RNA CTBP1 antisense RNA 2, microRNA-141-3p, ubiquitin-specific protease 22, long non-coding RNAs

## Abstract

Long non-coding RNAs (lncRNAs) feature prominently in pancreatic carcinoma progression. The present study aimed to clarify the biological functions, clinical significance and underlying mechanism of lncRNA CTBP1 antisense RNA 2 (CTBP1-AS2) in pancreatic carcinoma. Reverse transcription-quantitative PCR was performed to assess the expression levels of CTBP1-AS2, microRNA (miR)-141-3p and ubiquitin-specific protease 22 (USP22) mRNA in pancreatic carcinoma tissues and cell lines. Western blotting was used to examine USP22 protein expression in pancreatic carcinoma cell lines. Loss-of-function experiments were used to analyze the regulatory effects of CTBP1-AS2 on proliferation, apoptosis, migration and invasion of pancreatic carcinoma cells. Dual-luciferase reporter assay was used to examine the binding relationship between CTBP1-AS2 and miR-141-3p, as well as between miR-141-3p and USP22. It was demonstrated that CTBP1-AS2 expression was markedly increased in pancreatic carcinoma tissues and cell lines. High CTBP1-AS2 expression was associated with advanced clinical stage and lymph node metastasis of patients. Functional experiments confirmed that knocking down CTBP1-AS2 significantly inhibited pancreatic carcinoma cell proliferation, migration and invasion, and promoted cell apoptosis. In terms of mechanism, it was found that CTBP1-AS2 adsorbed miR-141-3p as a molecular sponge to upregulate the expression level of USP22. In conclusion, lncRNA CTBP1-AS2 may be involved in pancreatic carcinoma progression by regulating miR-141-3p and USP22 expressions; in addition, CTBP1-AS2 may be a diagnostic biomarker and treatment target for pancreatic carcinoma.

## Introduction

Known as a common malignancy in the digestive system, pancreatic carcinoma is a deadly disease. The incidence rates in males and females were 14.9 and 11.6 cases per 100,000 people annually in 2013–2017, and the mortality rates were 12.7 and 9.6 deaths per 100,000 people annually, respectively (1). Although great progress has been made in surgical resection, radiotherapy and chemotherapy (2,3), the five-year overall survival rate for patients with pancreatic carcinoma is only 8.3% (4). There is an urgent requirement to find new diagnostic markers and therapeutic targets for pancreatic carcinoma.

In recent years, novel functional molecules in cancer biology have been identified. Among them, long non-coding RNAs (lncRNAs) have been demonstrated to be crucial regulators in tumorigenesis and cancer progression. LncRNAs have >200 nucleotides but possess no protein-coding ability (5). LncRNAs have important biological functions and they participate in regulating tumor cell growth, migration, invasion and other malignant biological behaviors (5). Reportedly, a number of lncRNAs have been shown to serve a role in pancreatic carcinoma development as cancer-promoting factors or tumor suppressors, such as DNAH17-AS1 (6), CASC2 (7) and SNHG16 (8). However, the roles and mechanisms of lncRNAs in pancreatic carcinoma remain to be elucidated.

CTBP1 antisense RNA 2 (CTBP1-AS2) is a newly discovered oncogenic lncRNA that is abnormally expressed in hepatocellular cancer (9) and endometrial cell cancer (10); however, its role in pancreatic carcinoma is unclear. Through the Gene Expression Profiling Interactive Analysis (GEPIA) database it was found that CTBP1-AS2 was significantly highly expressed in pancreatic carcinoma tumor tissues, suggesting that it may have important regulatory functions in the tumorigenesis of pancreatic cancer. The present study aimed to analyze the effects of CTBP1-AS2 on pancreatic carcinoma cell proliferation, apoptosis, migration and invasion and to decipher the molecular mechanism of CTBP1-AS2 in pancreatic carcinoma progression. The present study, for the first time, to the best of our knowledge, elucidated the functions of CTBP1-AS2 in pancreatic carcinoma and provided a novel therapeutic target for this disease.

## Materials and methods

### Tissue sample collection

The present study followed the Declaration of Helsinki and was endorsed by the Ethics Committee of the People's Hospital of Ningxia Hui Autonomous Region (approval no. 2021-10-003). The human tissue samples were obtained from the Human Tissue Bank of People's Hospital of Ningxia Hui Autonomous Region. All patients signed the informed consent before surgery. Tumor tissues and para-cancerous tissues (≥3 cm away from the tumor margin) of 30 patients with pancreatic carcinoma (age range, 53–68 years; 14 male and 16 female) between October2021 and November 2012 were collected during surgery from the People's Hospital of Ningxia Hui Autonomous Region and immediately stored in liquid nitrogen at −196°C. None of the subjects had undergone chemotherapy or radiotherapy prior to the surgery.

### Cell culture

Normal human pancreatic duct epithelial cell line (HPDE6-C7), human pancreatic carcinoma cell lines (Hs766T, SW1990, CAPAN-1) and 293T were obtained from the American Type Culture Collection; the JF305 pancreatic carcinoma cell line was purchased from Yuchi (Shanghai) Biological Technology Co., Ltd. (cat. no. SCO423). All the cells were cultured in RPMI-1640 medium (Invitrogen; Thermo Fisher Scientific, Inc.) containing 10% fetal bovine serum (FBS; Invitrogen; Thermo Fisher Scientific, Inc.) in 5% CO_2_ at 37°C.

### Cell transfection

Hs766T and JF305 cells were inoculated into 6-well plates at a density of 2×10^5^ cells/well. Small interfering (si)RNA oligonucleotides targeting CTBP1-AS2 (si-CTBP1-AS2-1, 5′-GAGATCTAAGAAAAAATTCCAGA-3′; si-CTBP1-AS2-2, 5′-GCGCGTTATCATGACTTCTATTT-3′), scrambled siRNA [negative control siRNA (si-NC); 5′-TTCTCCGAACGTGTCACGTTT-3′], miR-141-3p mimic (5′-UAACACUGUCUGGUAAAGAUGG-3′), miR-141-3p inhibitor (5′-CCAUCUUUACCAGACAGUGUUA-3′), mimics NC (5′-UCACAACCUCCUAGAAAGAGUAGA-3′), inhibitor NC (5′-CAGUACUUUUGUGUAGUACAAA-3′), pcDNA3.1 vector overexpressing ubiquitin-specific protease 22 (USP22) and empty plasmids were provided by Guangzhou RiboBio Co., Ltd. The transfection was performed by Lipofectamine^®^ 2000 (Invitrogen; Thermo Fisher Scientific, Inc.) according to the manufacturer's instruction at 37°C and 5% CO_2_ for 6 h, with final concentrations of 50 nM siRNAs, miRNA mimics and inhibitor, and 2 µg overexpression plasmid. Finally, cells were incubated at 37°C for 48 h and harvested for the following study.

### Reverse transcription-quantitative polymerase chain reaction (RT-qPCR)

TRIzol^®^ (Thermo Fisher Scientific, Inc.) was used to extract lncRNA or mRNA from cells (5×10^5^) according to the manufacturer's instructions. A mirVana miRNA isolation kit (Ambion; Thermo Fisher Scientific, Inc.) was used to extract miRNA from tissues and cell lines according to the manufacturer's instructions. cDNA synthesis was conducted using the Mir-X miRNA First-Strand Synthesis kit (Takara Biotechnology Co., Ltd.) for miR-141-3p and a PrimeScript RT reagent kit (Promega Corporation) was adopted for CTBP1-AS2 and USP22 according to the manufacturer's instructions. An ABI 7500 Real-Time PCR System (Applied Biosystems; Thermo Fisher Scientific, Inc.) was used for performing qPCR with a SYBR Green Premix Ex Taq kit (Takara Biotechnology Co., Ltd.) according to the manufacturer's instructions. The reaction conditions were as follows: 95°C for 10 min; followed by 40 cycles of 95°C for 15 sec, 60°C for 30 sec and 72°C for 30 sec. GAPDH and U6 served as internal references, and the 2^−ΔΔCq^ method (11) was used for calculating the data. Primer sequences are listed in Table I.

### Cell Counting Kit-8 (CCK-8) assay

Pancreatic carcinoma cells were seeded into 96-well plates (2×10^3^ cells/well) and cultured for 0, 24, 36 and 72 h. At each time point 10 µl of CCK-8 solution (5 mg/ml; Beyotime Institute of Biotechnology) was added, and the cells were incubated for an additional 4 h. A microplate reader (Thermo-Fisher Scientific, Inc.) was used to measure the absorbance at 450 nm, which indicated the viability of the cells.

### 5-ethynyl-2-deoxyuridine (EdU) incorporation assay

An EdU Labeling/Detection kit (Guangzhou RiboBio Co., Ltd.) was used. The cells were inoculated into 96-well plates (5×10^3^ cells/well) for 24 h and then incubated with 50 mmol/l EdU solution in 5% CO_2_ at 37°C for 2 h. The cells were then fixed for 30 min at room temperature with 4% paraformaldehyde and incubated with glycine for 10 min at room temperature. Next, 100 µl of PBS containing 0.5% TritonX-100 was added into each well and the cells were incubated for 10 min at room temperature. Then the cells were washed twice with PBS and stained with 1X Apollo fluorochrome at room temperature for 30 min in the dark. Next, the cell nuclei were stained by 1X DAPI staining solution for 15 min at room temperature. A Nikon Eclipse Ti fluorescence microscope (Nikon Corporation) was used to observe the cells and to determine the percentage of EdU-positive cells, which indicated the proliferative capacity.

### Transwell assay

Transwell chambers (24-well; pore size of 8 µm; Corning, Inc.) were used to evaluate cell invasion and migration. Briefly, for the migration assay, pancreatic carcinoma cells in 200 µl FBS-free RPMI-1640 medium were transferred into the upper compartment (1×10^5^ cells/well) and the lower compartment contained 600 µl of RPMI-1640 medium containing 10% FBS. After 24 h, the cells in the upper compartment were removed, the cells on the lower surface of the filter were stained with 0.1% crystal violet for 30 min at room temperature and the number of migrated cells was counted under a microscope (Olympus Corporation). The cell invasion assay was carried out following the same procedures after the filter was precoated with 60 µl of Matrigel at 37°C for 2 h (BD Biosciences).

### Flow cytometric analysis

Apoptosis was examined using an Annexin V-FITC Apoptosis Detection kit (Beyotime Institute of Biotechnology, Inc.). Briefly, 48 h after transfection, cells were harvested, resuspended in binding buffer and incubated with 5 µl of Annexin V-FITC and 10 µl of PI in the dark at room temperature for 15 min. The apoptotic rate (early + late apoptotic cells) were analyzed by flow cytometry using an Attune NxT flow cytometer (Thermo Fisher Scientific, Inc.) and FlowJo software (version 10.0; BD Biosciences) (12).

### Luciferase reporter gene assay

Wild-type (WT) and mutant-type (MUT) CTBP1-AS2 luciferase reporter plasmids (CTBP1-AS2-WT and CTBP1-AS2-MUT) and USP22 3′UTR luciferase reporter plasmids (USP22-3′UTR-WT and USP22-3′UTR MUT) were obtained from Shanghai GenePharma Co., Ltd. Subsequently, these luciferase reporters were co-transfected with either miR-141-3p mimics or mimics NC into 293T cell line (1×10^5^ cells/well) by using Lipofectamine^®^ 2000 (Invitrogen; Thermo Fisher Scientific, Inc.) at 37°C. After 48 h, the luciferase activity of the cells was detected by the Dual-Luciferase Reporter Assay System (Promega Corporation). Firefly luciferase activity was normalized to *Renilla* luciferase activity.

### Western blot analysis

Proteins were extracted from pancreatic carcinoma tissues and cell lines using RIPA lysis buffer (Sigma-Aldrich; Merck KGaA), and a BCA Protein Assay kit (Thermo Fisher Scientific, Inc.) was used for protein quantification. Proteins (30 µg/lane) were separated using 10% SDS-PAGE (Sigma-Aldrich; Merck KGaA) and then transferred to polyvinylidene difluoride membranes (MilliporeSigma). After being blocked with 5% skimmed milk at 4°C for 2 h, the membranes were incubated with primary antibodies (anti-USP22; 1:2,000; cat. no. LS-C102769; LifeSpan BioSciences, Inc.; anti-GAPDH; 1:1,000; ab9385; Abcam) at 4°C overnight and then incubated with a HRP-conjugated secondary antibody (1:10,000; ab6721; Abcam) for 1 h at 37°C. Amersham ECL Select Western Blotting Detection Reagent (Cytiva) was used to visualize the protein bands. ImageJ software 1.8.0 (National Institutes of Health) was used to quantify the protein bands.

### Bioinformatics analysis using GEPIA and StarBase

The GEPIA database (http://gepia.cancer-pku.cn) is an interactive web server for tumor gene expression analysis by using data from The Cancer Genome Atlas Pan-Cancer project and Genotype-Tissue Expression database. The GEPIA database was searched to analyze the expression of CTBP1-AS2 in pancreatic carcinoma tissues and normal pancreatic tissues. StarBase database (http://starbase.sysu.edu.cn) was used to search for the miRNAs with potential binding sites for CTBP1-AS2 and mRNAs that bind to miR-141-3p.

### Statistical analysis

Each experiment was conducted at least three times, and all data are expressed as mean ± standard deviation or median ± interquartile range. SPSS 19.0 software (IBM Corp.) was used for statistical analysis. Data were analyzed using unpaired or paired Student's t-test, one-way ANOVA with Tukey's post hoc test, Pearson's correlation analysis and χ^2^ test. P<0.05 was considered to indicate a statistically significant difference.

## Results

### CTBP1-AS2 expression is significantly elevated in pancreatic carcinoma tissues and is associated with pathological characteristics

**Figure 1.**
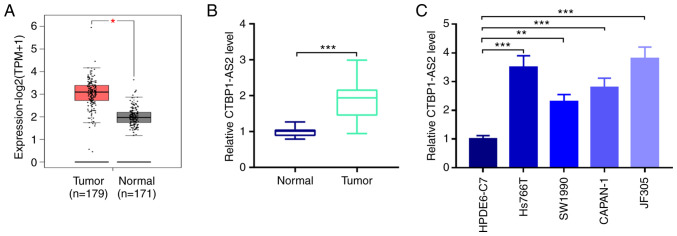
CTBP1-AS2 expression is significantly upregulated in pancreatic carcinoma. (A) The Gene Expression Profiling Interactive Analysis database was used to analyze the expression of CTBP1-AS2 in tumor tissues of patients with pancreatic carcinoma and normal pancreatic tissues. (B) RT-qPCR was used to detect CTBP1-AS2 expression in tumor tissues and adjacent normal tissues of patients with pancreatic carcinoma. (C) RT-qPCR was used to detect CTBP1-AS2 expression in normal human pancreatic duct epithelial cell line (HPDE6-C7) and pancreatic carcinoma cell lines. *P<0.05 vs. Normal, **P<0.01 vs. HPDE6-C7 and ***P<0.001 vs. Normal or HPDE6-C7. CTBP1-AS2, CTBP1 antisense RNA 2; RT-qPCR, reverse transcription-quantitative PCR; TPM, transcripts per kilobase of exon model per million mapped reads.

By searching the GEPIA database it was found that CTBP1-AS2 expression in patients with pancreatic carcinoma tumor tissues was significantly higher compared with that in normal pancreatic tissues (Fig. 1A). Similarly, RT-qPCR showed that CTBP1-AS2 expression was significantly higher in patient tumor tissues compared with para-carcinoma tissues (Fig. 1B). Additionally, compared with HPDE6-C7 normal pancreatic duct epithelial cells, CTBP1-AS2 expression was significantly higher in pancreatic carcinoma cell lines, Hs766T, SW1990, CAPAN-1 and JF305 (Fig. 1C). Since CTBP1-AS2 exhibited the highest expression level in Hs766T and JF305 cells, these two cell lines were selected to perform the subsequent loss-of-function experiments.

To analyze the clinical significance of high CTBP1-AS2 expression in pancreatic carcinoma, the relationship between patient clinicopathological characteristics and CTBP1-AS2 expression levels were evaluated (Table II). Based on the median value of CTBP1-AS2 expression level, 30 patients were divided into high and low CTBP1-AS2 expression groups (n=15 patients/group). The results showed that high CTBP1-AS2 expression was associated with lymph node metastasis and advanced clinical stage of patients with pancreatic carcinoma. Together, these data indicated that CTBP1-AS2 may be a crucial regulatory factor for pancreatic carcinoma development and may serve a cancer-promoting role.

### CTBP1-AS2 promotes pancreatic carcinoma cell proliferation, migration and invasion and inhibited cell apoptosis

**Figure 2.**
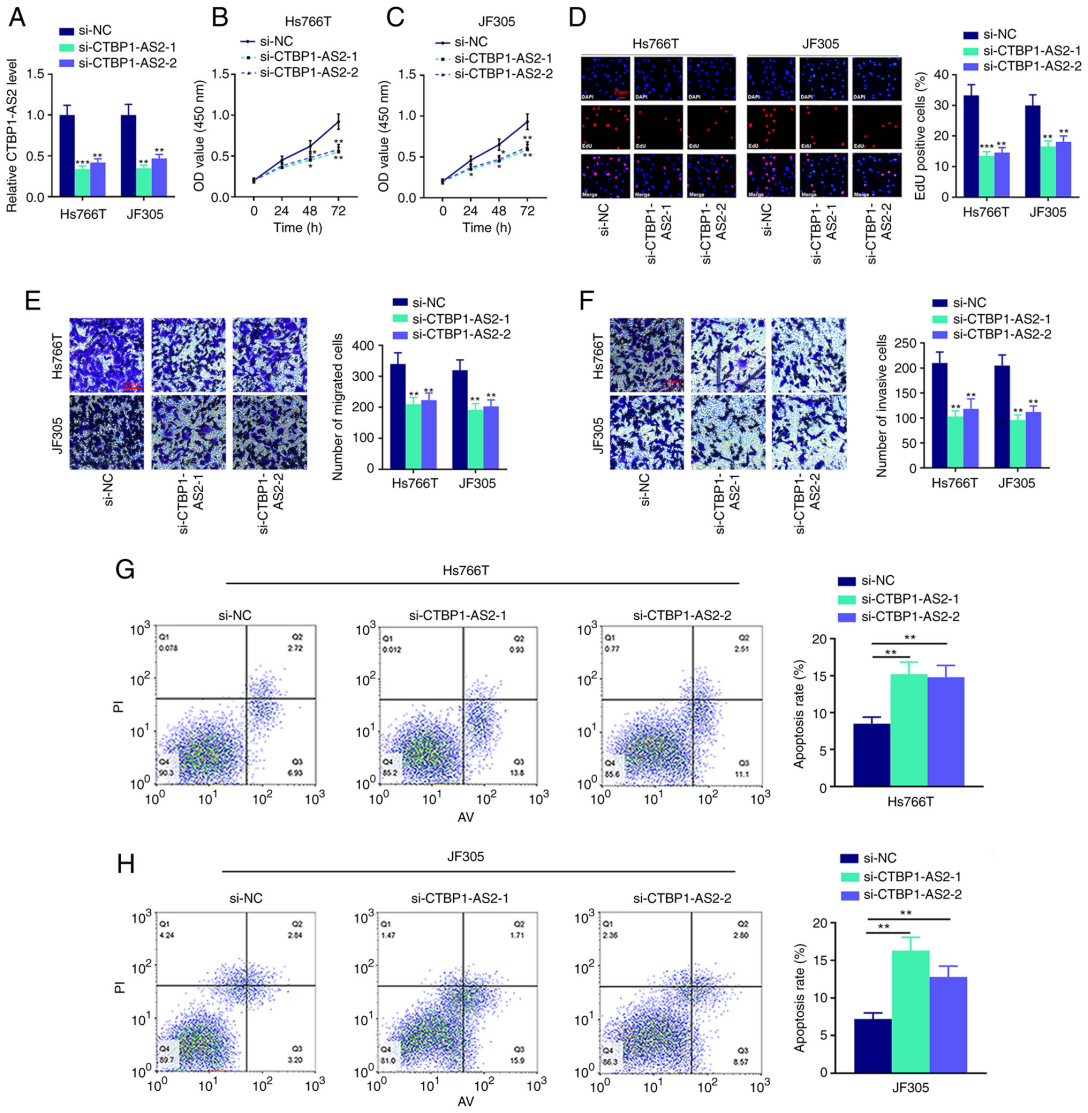
CTBP1-AS2 knockdown suppresses pancreatic carcinoma cell proliferation, migration and invasion and induces cell apoptosis. (A) CTBP1-AS2 siRNAs were transfected into Hs766T and JF305 cell lines, and the transfection efficiency was verified by reverse transcription-quantitative PCR. CCK-8 assay was used for examining the proliferation of (B) Hs766T and (C) JF305 cell lines transfected with CTBP1-AS2 siRNAs. (D) EdU assay was used to examine the proliferation of Hs766T and JF305 cell lines following the transfection with CTBP1-AS2 siRNA. Transwell and Matrigel assays were used to detect the (E) migratory and (F) invasive abilities, respectively, of Hs766T and JF305 cell lines following si-CTBP1-AS2 transfection. Flow cytometry was used to detect the apoptosis rate of (G) Hs766T and (H) JF305 cell lines following the transfection with CTBP1-AS2 siRNA. *P<0.05, **P<0.01 and ***P<0.001 vs. si-NC. CTBP1-AS2, CTBP1 antisense RNA 2; EdU, 5-ethynyl-2-deoxyuridine; NC, negative control; si, short interfering RNA.

To examine the biological functions of CTBP1-AS2 in pancreatic carcinoma cells, two CTBP1-AS2 siRNAs (si-CTBP1-AS2-1 and si-CTBP1-AS2-2) were used to knock down CTBP1-AS2 expression in Hs766T and JF305 cell lines. It was demonstrated that CTBP1-AS2 expression levels in both CTBP1-AS2 knockdown groups were significantly reduced compared with the si-NC group (Fig. 2A). CCK-8 and EdU assays confirmed that the cell proliferation was significantly inhibited after CTBP1-AS2 knockdown (Fig. 2B-D). Transwell and Matrigel assays showed that cell migration and invasion, respectively, were notably suppressed after CTBP1-AS2 knockdown (Fig. 2E and F). It was also revealed that Hs766T and JF305 cells transfected with si-CTBP1-AS2 had higher apoptosis rates compared with cells transfected with si-NC (Fig. 2G and H). These results suggested that knocking down CTBP1-AS2 could inhibit the malignant phenotypes of pancreatic carcinoma cells.

### CTBP1-AS2 targets miR-141-3p

**Figure 3.**
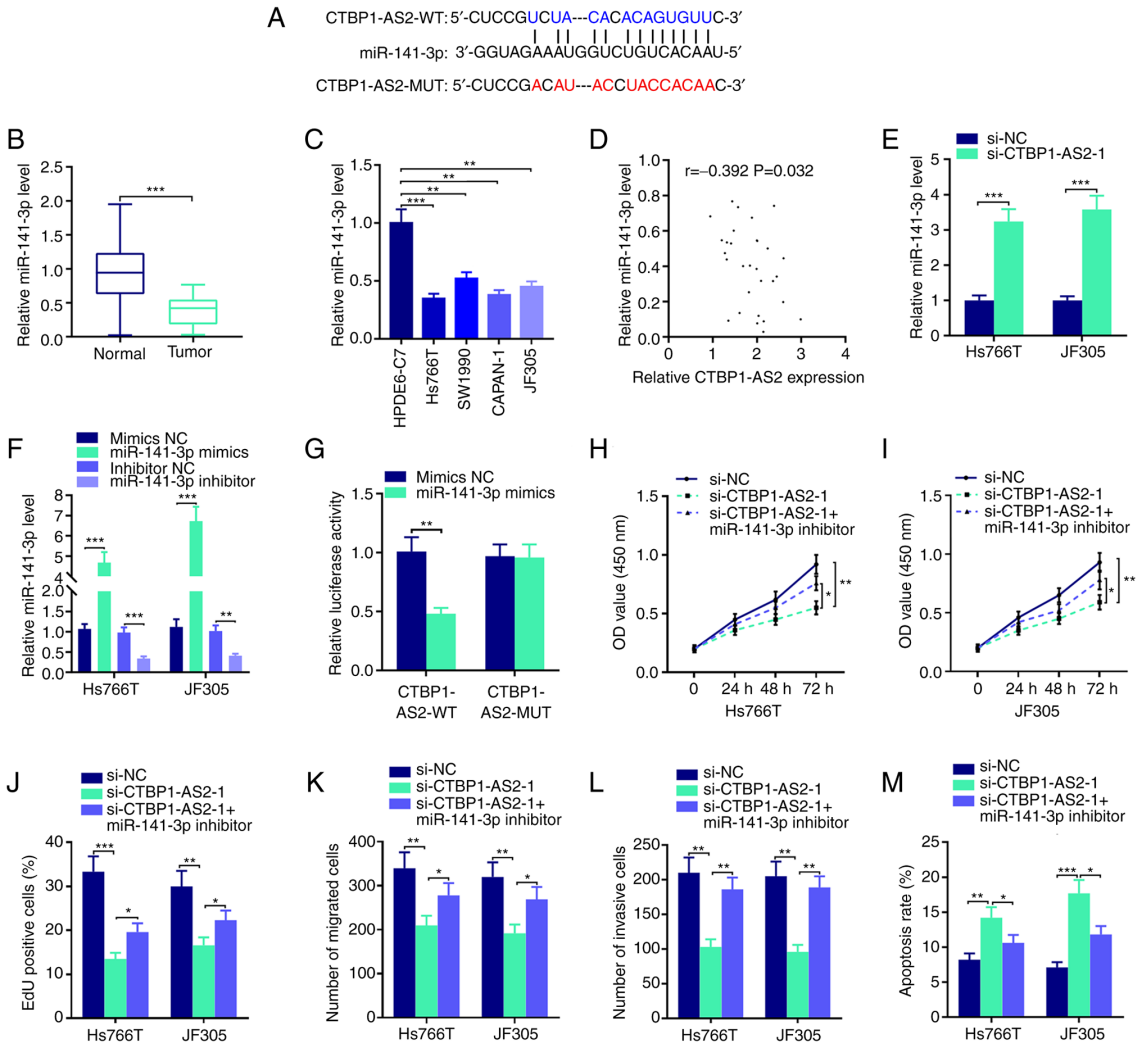
CTBP1-AS2 targets miR-141-3p to promote pancreatic carcinoma progression. (A) StarBase database showed that CTBP1-AS2 sequence contained the binding site complementary to miR-141-3p. RT-qPCR was performed to detect the expression level of miR-141-3p in pancreatic carcinoma (B) tissues and (C) cell lines. (D) Pearson's correlation analysis of CTBP1-AS2 and miR-141-3p expression levels in pancreatic carcinoma tissues. (E) RT-qPCR was used to examine miR-141-3p expression levels in Hs766T and JF305 cell lines following the transfection of siCTBP1-AS2-1. (F) RT-qPCR was used to examine miR-141-3p expression in Hs766T and JF305 cell lines following the transfection of miR-141-3p mimics or miR-141-3p inhibitor. (G) Dual-luciferase reporter assay was used to verify the inaction between miR-141-3p and CTBP1-AS2. CCK-8 assay was conducted to examine proliferation of (H) Hs766T and (I) JF305 cell lines following the co-transfection of si-CTBP1-AS2-1 and miR-141-3p inhibitor. (J) EdU assay was also used to examine proliferation in Hs766T and JF305 cell line following the co-transfection of si-CTBP1-AS2-1 and miR-141-3p inhibitor. Transwell and Matrigel assays were performed to detect (K) migratory and (L) invasive abilities, respectively, of Hs766T and JF305 cell lines following the co-transfection of si-CTBP1-AS2-1 and miR-141-3p inhibitor. (M) Flow cytometry was performed to detect the apoptosis of Hs766T and JF305 cells following the co-transfection of si-CTBP1-AS2-1 and miR-141-3p inhibitor. *P<0.05 vs. si-CTBP1-AS2-1, **P<0.01 vs. HPDE6-C7, inhibitor NC, mimics NC, si-NC or si-CTBP1-AS2-1. ***P<0.001 vs. Normal, HPDE6-C7, si-NC or mimics NC. CTBP1-AS2-1, CTBP1 antisense RNA 2 siRNA-1; EdU, 5-ethynyl-2-deoxyuridine; miR, microRNA; MUT, mutant; NC, negative control; RT-qPCR, reverse transcription-quantitative PCR; si, short interfering RNA; WT, wild-type.

LncRNAs can take part in tumor progression as competing endogenous RNAs (ceRNAs), which means that lncRNAs can adsorb miRNAs like molecular sponges to regulate the expression levels of mRNAs (13). To further pinpoint the mechanism by which CTBP1-AS2 serves a role in pancreatic carcinoma, StarBase database was searched and it was found that CTBP1-AS2 likely binds with miR-141-3p (Fig. 3A). RT-qPCR results demonstrated that miR-141-3p expression was significantly reduced in tumor tissues and pancreatic carcinoma cell lines compared with that in the respective para-cancerous tissues and normal human pancreatic duct epithelial cell line (Fig. 3B and C). Furthermore, CTBP1-AS2 expression in pancreatic carcinoma tumor tissues was inversely correlated with miR-141-3p expression (Fig. 3D), and CTBP1-AS2 knockdown markedly promoted miR-141-3p expression in Hs766T and JF305 cell lines compared with si-NC (Fig. 3E). Subsequently, miR-141-3p expression was overexpressed or knocked down in Hs766T and JF305 cells by transfecting with miR-141-3p mimics or miR-141-3p inhibitor, respectively. The results showed that miR-141-3p mimics significantly increased the expression of miR-141-3p compared with mimics NC and miR-141-3p inhibitor decrease the miR-141-3p expression compared with the inhibitor NC (Fig. 3F). No significant difference in miR-141-3p expression was observed between mimic NC and inhibitor NC, so mimic NC was selected as the control of miR-141-3p mimics or miR-141-3p inhibitor in the following study. Dual-luciferase reporter assay results showed that miR-141-3p mimics could significantly reduce CTBP1-AS2-WT luciferase activity but failed to affect the luciferase activity of CTBP1-AS2-MUT, which verified the targeting relationship between CTBP1-AS2 and miR-141-3p (Fig. 3G). To determine whether CTBP1-AS2 could serve a role in pancreatic carcinoma through miR-141-3p, rescue assays were performed. The results showed that the effects of CTBP1-AS2 knockdown on pancreatic carcinoma cell proliferation, migration, invasion and apoptosis could be partially counteracted by inhibiting miR-141-3p (Fig. 3H-M and Fig. S1). These results displayed that CTBP1-AS2 exerted a cancer-promoting effect by repressing miR-141-3p expression in pancreatic carcinoma.

### miR-141-3p directly targets USP22

**Figure 4.**
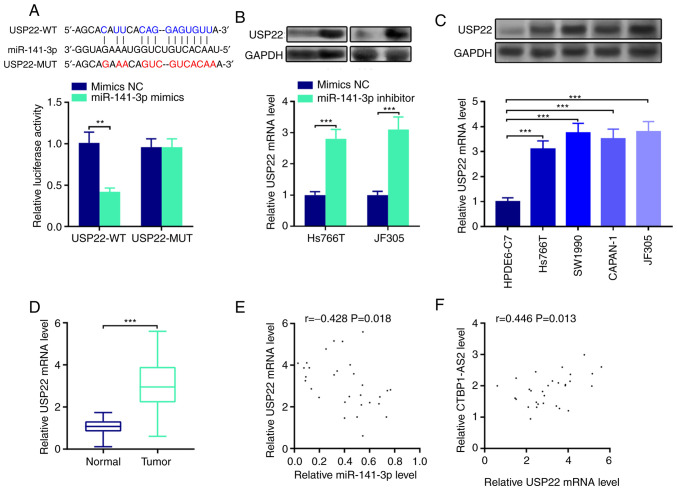
miR-141-3p directly targets USP22. (A) StarBase database search indicated that the 3′UTR of USP22 mRNA contained a target site for miR-141-3p, which was confirmed by luciferase reporter assay. (B) Western blotting (top) and RT-qPCR (bottom) were conducted to detect the expression levels of USP22 protein and mRNA, respectively, in pancreatic carcinoma cells transfected with miR-141-3p inhibitor. (C) Western blotting (top) and RT-qPCR (bottom) were used to detect USP22 protein and mRNA expression, respectively, in pancreatic carcinoma cell lines. (D) RT-qPCR was used to detect USP22 expression in pancreatic tumor tissues. Pearson's correlation analysis of (E) USP22 mRNA and miR-141-3p expression levels and (F) USP22 mRNA and CTBP1-AS2 expression levels in pancreatic carcinoma tissues. **P<0.01 vs. mimics NC and ***P<0.001 vs. mimics NC, HPDE6-C7 or Normal. CTBP1-AS2, CTBP1 antisense RNA 2; miR, microRNA; MUT, mutant; RT-qPCR, reverse transcription-quantitative PCR; NC, negative control; USP22, ubiquitin-specific protease 22; WT, wild-type.

Putative downstream mRNAs interacting with miR-141-3p were also investigated. StarBase database analysis identified a number of potential targets of miR-141-3p, such as E2F transcription factor 3, cyclin G1 and ring finger protein 38. Among them, USP22 was selected in a previous study which demonstrated that USP22 served as a cancer-promoting gene in the progression of pancreatic cancer (14) (Fig. 4A). Results from the dual-luciferase reporter assay showed that miR-141-3p mimics inhibited USP22-WT luciferase activity but did not change USP22-MUT luciferase activity (Fig. 4A). In addition, miR-141-3p inhibitor was transfected into Hs766T and JF305 cell lines and it was found that USP22 mRNA and protein expression levels were significantly decreased following the transfection compared with the control group (Fig. 4B). Notably, USP22 expression levels were significantly enhanced in pancreatic carcinoma cell lines and tumor tissues (Fig. 4C and D, respectively). miR-141-3p expression was negatively correlated with USP22 mRNA expression (Fig. 4E), and CTBP1-AS2 expression was positively correlated with USP22 mRNA expression in pancreatic carcinoma tissues (Fig. 4F). These findings revealed that miR-141-3p targeted and downregulate USP22 expression in pancreatic carcinoma.

### CTBP1-AS2 upregulates USP22 expression through miR-141-3p inhibition

**Figure 5.**
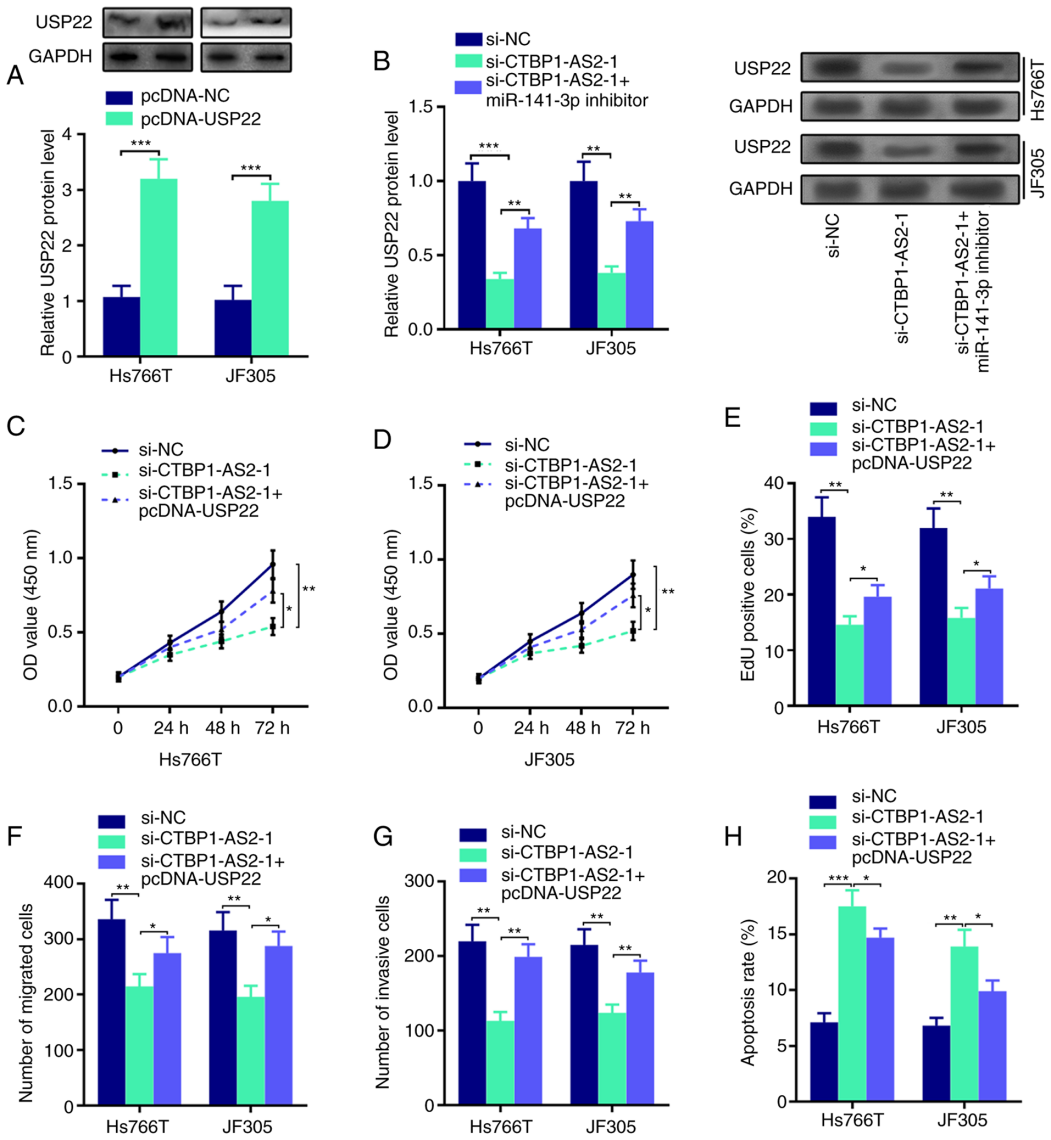
CTBP1-AS2 upregulates USP22 expression. (A) Western blotting was performed to examine USP22 protein expression in Hs766T and JF305 cell lines following the transfection of USP22 overexpression plasmid. (B) Western blotting was performed to examine USP22 protein expression levels in Hs766T and JF305 cells following the co-transfection of si-CTBP1-AS2-1 and miR-141-3p inhibitor. CCK-8 assay was conducted to evaluate proliferation in (C) Hs766T and (D) JF305 cells following the co-transfection of si-CTBP1-AS2-1 and USP22 overexpression plasmid. (E) EdU assay was also used to detect proliferation of Hs766T and JF305 cells following the co-transfection of si-CTBP1-AS2-1 and USP22 overexpression plasmid. Transwell and Matrigel assays were used to detect (F) migratory and (G) invasive abilities, respectively, in Hs766T and JF305 cells following the co-transfection of si-CTBP1-AS2-1 and USP22 overexpression plasmid. (H) Flow cytometry was used to detect the apoptosis of Hs766T and JF305 cells following the co-transfection of si-CTBP1-AS2-1 and USP22 overexpression plasmid. *P<0.05 vs. si-CTBP1-AS2-1, **P<0.01 vs. si-CTBP1-AS2-1 or si-NC and ***P<0.001 vs. pcDNA-NC or si-NC. CTBP1-AS2-1, CTBP1 antisense RNA 2 siRNA-1; EdU, 5-ethynyl-2-deoxyuridine; miR, microRNA; pcDNA, pcDNA3.1 vector; si, short interfering RNA; USP22, ubiquitin-specific protease 22.

Western blot assay results showed that USP22 protein expression was upregulated in Hs766T and JF305 cells transfected with USP22 overexpression plasmid compared with the control (Fig. 5A). In addition, the inhibitory effects of CTBP1-AS2 knockdown on USP22 protein expression could be abolished by co-transfection with miR-141-3p inhibitor (Fig. 5B). In addition, the transfection of USP22 overexpression plasmid could reverse CTBP1-AS2 knockdown-mediated inhibitory effects on the proliferation, migration and invasion of Hs766T and JF305 cells (Fig. 5C-H; Fig. S2). These results suggested that CTBP1-AS2 may serve a cancer-promoting role through regulating the miR-141-3p/USP22 axis.

## Discussion

The morbidity and mortality rates of pancreatic carcinoma both increased by an average of 0.3% per year during the past decade (1), so it is necessary to clarify the mechanism of pancreatic carcinoma tumorigenesis to find new therapeutic targets (15). The present study demonstrated that CTBP1-AS2 expression was upregulated in pancreatic carcinoma tissues and cell lines, and high CTBP1-AS2 expression was associated with lymph node metastasis and the advanced clinical stage of patients with pancreatic carcinoma. CTBP1-AS2 expression increased pancreatic carcinoma cell proliferation, migration and invasion and repressed apoptosis by regulating miR-141-3p/USP22 axis.

LncRNAs participate in the pathogenesis of a number of cancers, including pancreatic carcinoma (16). Previous studies have reported the role of CTBP1-AS2 in cancers and other diseases. Specifically, CTBP1-AS2 can facilitate hepatocellular carcinoma cell proliferation by regulating the miR-623/Cyclin D1 axis (9). In gastric cancer, CTBP1-AS2 promotes cells proliferation and metastasis and suppresses apoptosis by regulating the miR-139-3p/MMP11 axis (17). CTBP1-AS2 expression is upregulated in osteoarthritis and increases the methylation of miR-130a gene to suppress the proliferation of chondrocytes (18). CTBP1-AS2 regulates the epithelial-mesenchymal transition (EMT) of glioma by modulating the miR-370-3p/Wnt7a axis (19). Additionally, in ovarian cancer, CTBP1-AS2 overexpression results in the reduced proliferation rate of cancer cells through the miR-216a/PTEN axis (20). In cervical cancer, CTBP1-AS2 facilitates cervical cancer progression by sponging miR-3163 to upregulate ZNF217 expression (21). The present study demonstrated that CTBP1-AS2 expression was significantly higher in pancreatic carcinoma tumor tissues compared with para-cancerous tissues, and high CTBP1-AS2 expression was associated with the unfavorable pathological indices of the patients. Functional experiments verified that knocking down CTBP1-AS2 could significantly inhibit pancreatic carcinoma cell proliferation, migration and invasion and induce cell apoptosis. These results displayed that CTBP1-AS2 may serve a tumor-promoting function in pancreatic carcinoma progression.

LncRNAs can adsorb miRNAs like sponges and therefore indirectly regulate downstream mRNA expression, by which they serve a role in regulating biological processes (13). There are also a number of lncRNA-miRNA-mRNA regulatory networks in pancreatic carcinoma. For instance, lncRNA DNAH17-AS1 upregulates PPME1 expression through decoying miR-432-5p to promote pancreatic carcinoma cell proliferation, migration and invasion (6). Another study reported that CASC2 induces the expression of PTEN to suppress pancreatic carcinoma cell metastasis by sponging miR-21 (7). The present study found that miR-141-3p was the target of CTBP1-AS2, whose expression could be negatively regulated by CTBP1-AS2. miR-141-3p expression is reported to be decreased and act as a tumor suppressor in several types of tumors. For example, miR-141-3p expression is downregulated in osteosarcoma; miR-141-3p targets FUS to degrade L-lactate dehydrogenase B chain to reduce the degree of malignancy of osteosarcoma (22). In addition, miR-141-3p suppresses colorectal cancer cell proliferation, migration and invasion by targeting TNF receptor-associated factor 5 (23). A recent study reported that in pancreatic carcinoma, miR-141-3p expression is significantly reduced (24), which is consistent with the findings of the present study, and that XIST upregulates TGF-β2 expression by targeting miR-141-3p, therefore promoting pancreatic carcinoma cell proliferation, migration and invasion. Another study demonstrated that miR-141-3p can directly target MAP4K4 to repress pancreatic carcinoma cell invasion (25). The present study showed that miR-141-3p expression in pancreatic carcinoma tissues was negatively correlated with CTBP1-AS2 expression and that inhibition of miR-141-3p counteracted the effects of CTBP1-AS2 knockdown on regulating pancreatic carcinoma cell proliferation, migration, invasion and apoptosis. It was suggested that CTBP1-AS2 may serve a role in pancreatic carcinoma by sponging miR-141-3p.

In the present study, USP22 was identified as one of the targets of miR-141-3p. USP22 is a member of the deubiquitinating enzyme family; it can regulate cell metabolism and cell cycle, and it is also involved in the pathogenesis of a number of human diseases (26). In gastric cancer, USP22 expression is significantly upregulated; it can promote cancer cell proliferation and inhibit apoptosis through regulating son of sevenless 1/RAS protein axis (27). In retinoblastoma, USP22 knockdown promotes the senescence and apoptosis of retinoblastoma cells by regulating telomerase reverse transcriptase/p53 pathway (28). Importantly, in pancreatic carcinoma, USP22 is overexpressed and promotes migration, invasion and EMT of pancreatic carcinoma cells through the focal adhesion kinase pathway (14). Furthermore, USP22 knockdown in pancreatic carcinoma cells can promote the infiltration of T cells and natural killer cells in the tumor microenvironment (29). The present study revealed that USP22 expression was markedly elevated in pancreatic carcinoma cell lines and tumor tissues. Notably, CTBP1-AS2 could upregulate USP22 expression by repressing miR-141-3p expression. The rescue assays showed that the inhibiting effects of CTBP1-AS2 knockdown on pancreatic carcinoma cell proliferation, migration, invasion and apoptosis could be reversed by USP22 overexpression. Collectively, these findings validated the hypothesis that CTBP1-AS2 could function as a ceRNA to facilitate pancreatic carcinoma cell proliferation, migration and invasion by adsorbing miR-141-3p and upregulating USP22 expression.

Collectively, the present study revealed that CTBP1-AS2 was an oncogenic lncRNA by regulating miR-141-3p/USP22 axis in pancreatic cancer. The current study may provide an improved understanding of the lncRNA-miRNA-mRNA ceRNA network in pancreatic cancer development. However, there were some limitations to the study. For example, the function of CTBP1-AS2/miR-141-3p/USP22 in pancreatic cancer needs to be verified with *in vivo* experiment. In addition, the downstream signaling pathway of USP22 in pancreatic cancer also needs to be investigated in the future.

## Supplementary Material

Supporting Data

## Figures and Tables

**Table I.** Primers used for reverse transcription-quantitative PCR.

Transcript	Primer sequences (5′-3′)
CTBP1-AS2	F: CAAGGGCACTCAAAGGGCTA
	R: CAGGCAGGCAAACACAGAAC
miR-141-3p	F: CGCAGTAACACTGTCTGGT
	R: GTCCAGTTTTTTTTTTTTTTTCCATCT
USP22	F: GGCGGAAGATCACCACGTAT
	R: TTGTTGAGACTGTCCGTGGG
GAPDH	F: TGCACCACCAACTGCTTAGC
	R: GGCATGGACTGTGGTCATGAG
U6	F: GCTTCGGCAGCACATATACTAAAAT
	R: CGCTTCACGAATTTGCGTGTCAT

CTBP1-AS2, CTBP1 antisense RNA 2; F, forward; miR, microRNA; R, reverse; USP22, ubiquitin-specific protease 22.

**Table II.** Relationship between CTBP1-AS2 expression and clinicopathological characteristics of patients with pancreatic carcinoma.

		CTBP1-AS2		
			
Characteristic	Cases (n=30)	High (n=15)	Low (n=15)	P-value
Age, years				
≥60	17	10	7	0.269
<60	13	5	8	
Sex				
Male	14	6	8	0.464
Female	16	9	7	
Clinical stage				
III–IV	14	10	4	0.028^a^
I–II	16	5	11	
Lymph node metastasis				
Yes	14	11	3	0.003^a^
No	16	4	12	

CTBP1-AS2, CTBP1 antisense RNA 2.

a P<0.05.

## Data Availability

The data generated in the present study may be requested from the corresponding author.
