# Supplementary material for: Long non-coding RNA CTBP1-AS2 upregulates USP22 to promote pancreatic carcinoma progression by sponging miR-141-3p
Source: Mol Med Rep. 2022 Jan 14;25(3):86. doi: 10.3892/mmr.2022.12602 (PMC8809116; doi:10.3892/mmr.2022.12602)

Figure S1. miR-141-3p inhibitor partially reverses the effects of knocking down CTBP1-AS2 in pancreatic carcinoma cells. (A) Representative images of EdU assays used for the data presented in Fig. 3J. (B) Representative images of Transwell migration (left) and Matrigel invasion (right) assay used for the data presented in Fig. 3K and L, respectively. (C) Representative images of flow cytometry scatterplots used for the apoptosis data presented in Fig. 3M. AV, annexin V; CTBP1-AS2-1, CTBP1 antisense RNA 2 siRNA-1; EdU, 5-ethynyl-2-deoxyuridine; miR, microRNA; NC, negative control; si, short interfering RNA.

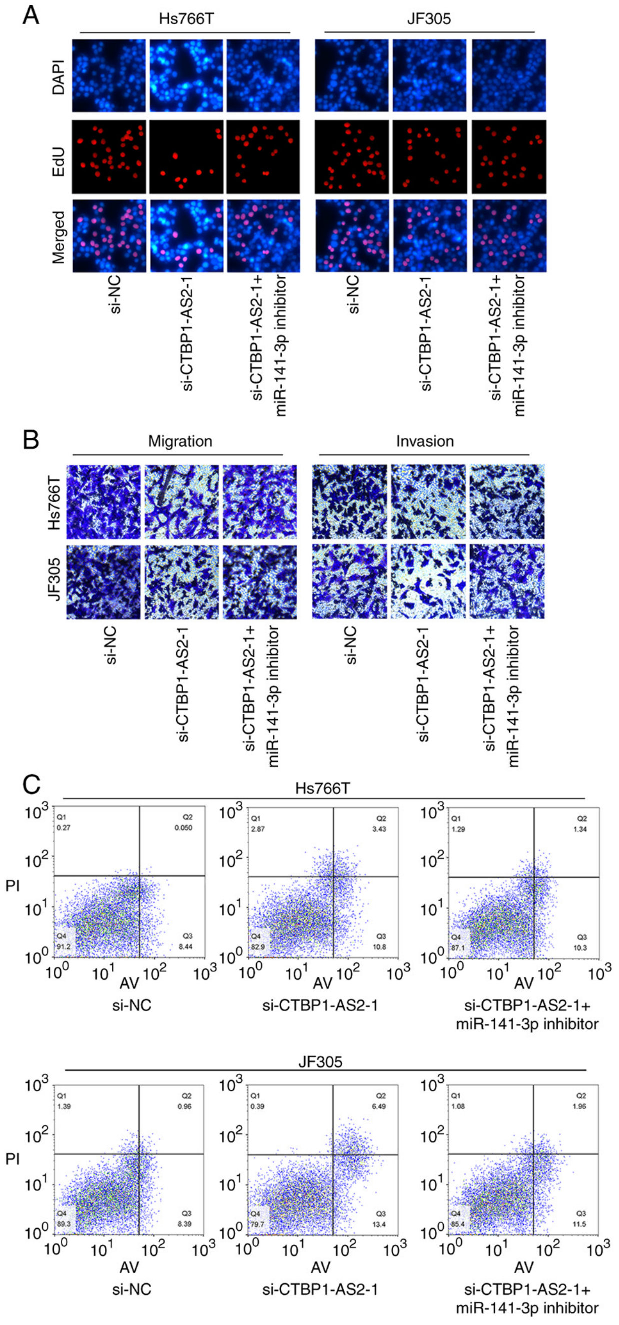

Figure S2. USP22 overexpression plasmid partially reverses the effects of knocking down CTBP1-AS2 in pancreatic carcinoma cells. (A) Representative images of EdU assay used for the data presented in Fig. 5E. (B) Representative images of Transwell migration (left) and Matrigel invasion (right) assays used for the data presented in Fig. 5F and G, respectively. (C) Representative images of flow cytometry scatterplots used for the apoptosis data presented in Fig. 5H. AV, annexin V; CTBP1-AS2-1, CTBP1 antisense RNA 2 siRNA-1; EdU, 5-ethynyl-2-deoxyuridine; miR, microRNA; NC, negative control; si, short interfering RNA.

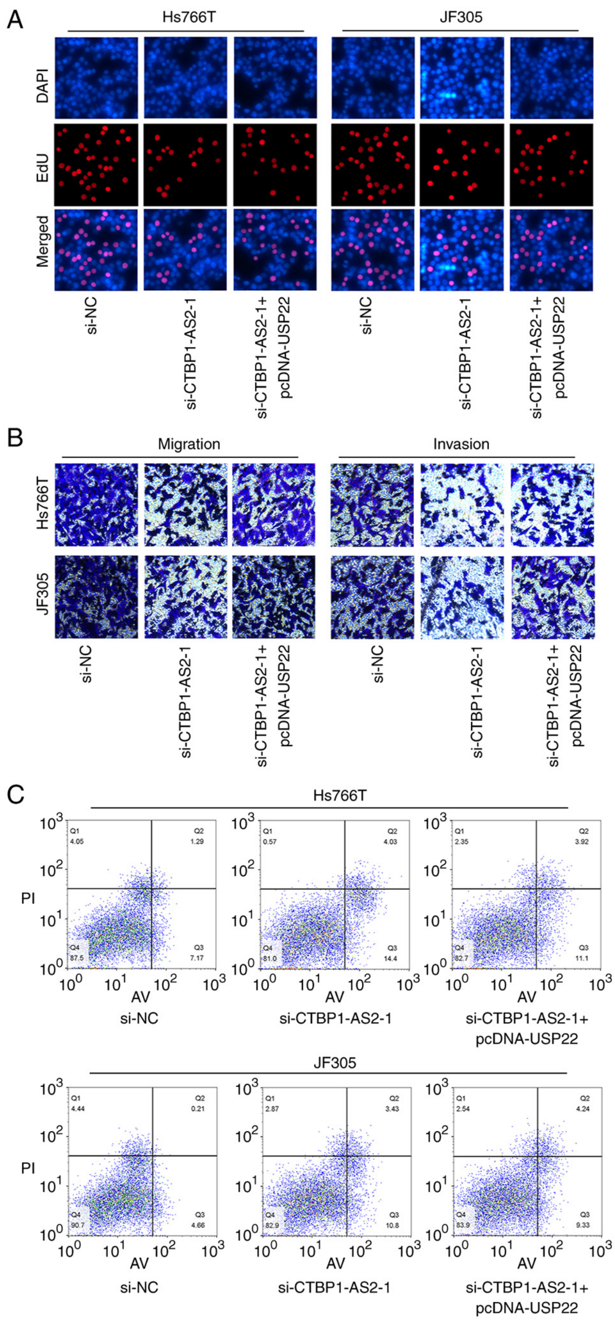

Supplement: Supporting Data [file Supplementary_Data.pdf]
